# Identification of Proteins Secreted by Malaria Parasite into Erythrocyte using SVM and PSSM profiles

**DOI:** 10.1186/1471-2105-9-201

**Published:** 2008-04-16

**Authors:** Ruchi Verma, Ajit Tiwari, Sukhwinder Kaur, Grish C Varshney, Gajendra PS Raghava

**Affiliations:** 1 Bioinformatics Centre, Institute of Microbial Technology, Sector 39-A, Chandigarh, India; 2 Cell biology and Immunology, Institute of Microbial Technology, Sector 39-A, Chandigarh, India

## Abstract

**Background:**

Malaria parasite secretes various proteins in infected RBC for its growth and survival. Thus identification of these secretory proteins is important for developing vaccine/drug against malaria. The existing motif-based methods have got limited success due to lack of universal motif in all secretory proteins of malaria parasite.

**Results:**

In this study a systematic attempt has been made to develop a general method for predicting secretory proteins of malaria parasite. All models were trained and tested on a non-redundant dataset of 252 secretory and 252 non-secretory proteins. We developed SVM models and achieved maximum MCC 0.72 with 85.65% accuracy and MCC 0.74 with 86.45% accuracy using amino acid and dipeptide composition respectively. SVM models were developed using split-amino acid and split-dipeptide composition and achieved maximum MCC 0.74 with 86.40% accuracy and MCC 0.77 with accuracy 88.22% respectively. In this study, for the first time PSSM profiles obtained from PSI-BLAST, have been used for predicting secretory proteins. We achieved maximum MCC 0.86 with 92.66% accuracy using PSSM based SVM model. All models developed in this study were evaluated using 5-fold cross-validation technique.

**Conclusion:**

This study demonstrates that secretory proteins have different residue composition than non-secretory proteins. Thus, it is possible to predict secretory proteins from its residue composition-using machine learning technique. The multiple sequence alignment provides more information than sequence itself. Thus performance of method based on PSSM profile is more accurate than method based on sequence composition. A web server PSEApred has been developed for predicting secretory proteins of malaria parasites,the URL can be found in the Availability and requirements section.

## Background

The human malaria caused by *Plasmodium falciparum *has been one of the major infectious diseases in the world causing illness in 300 to 600 million people leading to 2 to 3 million deaths annually [1]. In addition, it is putting huge economic burden on affected countries particularly in Asian and African subcontinents. In order to develop effective drugs and vaccines against this parasite it is important to identify novel potential drug/vaccine targets. Parasite secretes an array of proteins within the host erythrocyte and beyond to facilitate its own survival within the host cell and for immunomodulation. These proteins secreted by parasite can serve as potential drug/vaccine targets. The identification of secretory proteins of *Plasmodium falciparum *has got limited success, since experimental identification of these proteins is rather difficult due to complex nature of parasite.

In silico prediction of secretory proteins is need of time in the era of genomics where thousands of genomes have been completely sequenced including those of *P. falciparum *(size 22.8 MB; 14 chromosomes and 5300 proteins) [2]. It has been shown in past that secretory proteins of eukaryotes have signal sequence at N-terminus, which can be used to predict its secretory nature. One of the commonly used programs for predicting secretory proteins of eukaryotes is TargetP [3]. Though TargetP is successful for eukaryotic protein but fails to predict known *P. falicparum *secretory proteins like PfEMP1. The reason of failure of TargetP for *P. falciparum *is due to its complex life-cycle that alternate between vertebrate and invertebrate. Thus it is not possible to use subcellular localization methods developed either for eukaryotes [4] or prokaryote [5] for localization of *P. falciparum *proteins. There is a need to develop organism specific methods [6]. Recently, two groups independently identified the signal (PEXEL) or motif (VTS) in secretory proteins of *P. falciparum *partly responsible for proteins export from parasite to erythrocyte [7,8]. However, a number of well known and experimentally documented secretory/erythrocyte membrane associated proteins lack these motifs, thus emphasizing the existence of multiple pathways that operate in parallel [9]. With the completion of Plasmodium genome sequence, the challenge is to combine experimental and bioinformatics tools in order to develop algorithm with high predictive value for secretory proteins of malaria parasite.

In general, two important reasons for failure of these motif based methods are; i) all secretory proteins do not necessarily have signal peptide particularly those secreted by non-classical pathways and ii) location of signal is not conserved in protein, since it may be found on either N-terminal or C-terminal or in middle of proteins [10]. In order to overcome these limitations several groups have developed methods based on amino acid composition or dipeptide composition of proteins [6,11,12]. Recently two web servers (Signal-CF and Signal-3L) have been developed, which provides key steps important for predicting secretory proteins [13,14]. Though composition based methods have been developed for eukaryotic or prokrayotic proteins but till date no method has been developed for *P. falciparum *specific proteins. It has been demonstrated in past that organism specific methods perform better than general methods [6]. Thus there is need to develop method especially for predicting secretory proteins of *P. falciparum*.

In this paper, we describe a method developed for predicting secretory proteins of malaria parasite. First, amino acid sequence of a protein has been converted into fixed length patterns by computing various type of composition like amino acid, dipeptides. Then machine-learning technique Support Vector Machine (SVM) has been used to discriminate secretory and non-secretory protein. For the first time in this study, evolutionary information has been used for predicting secretory proteins. The evolutionary information in form of PSSM profile was obtained from PSI-BLAST search against "nr" databases. A web server has also been developed for predicting secretory proteins of malaria parasite.

## Results

### Analysis of amino acid composition

**Figure 1:**
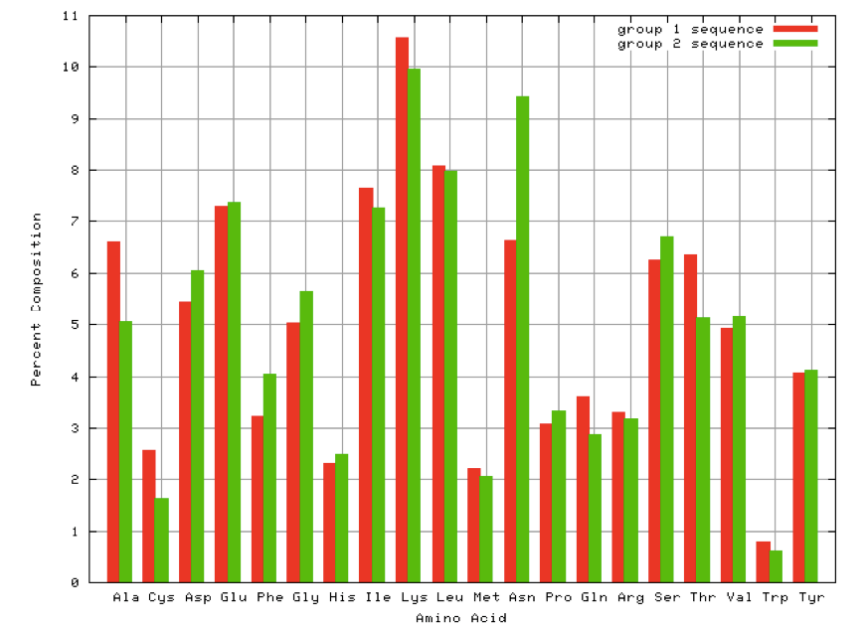
**Amino acid composition chart of secretory and non-secretory proteins.** Red and green lines represent the secretory and non-secretory proteins respectively.

**Figure 2:**
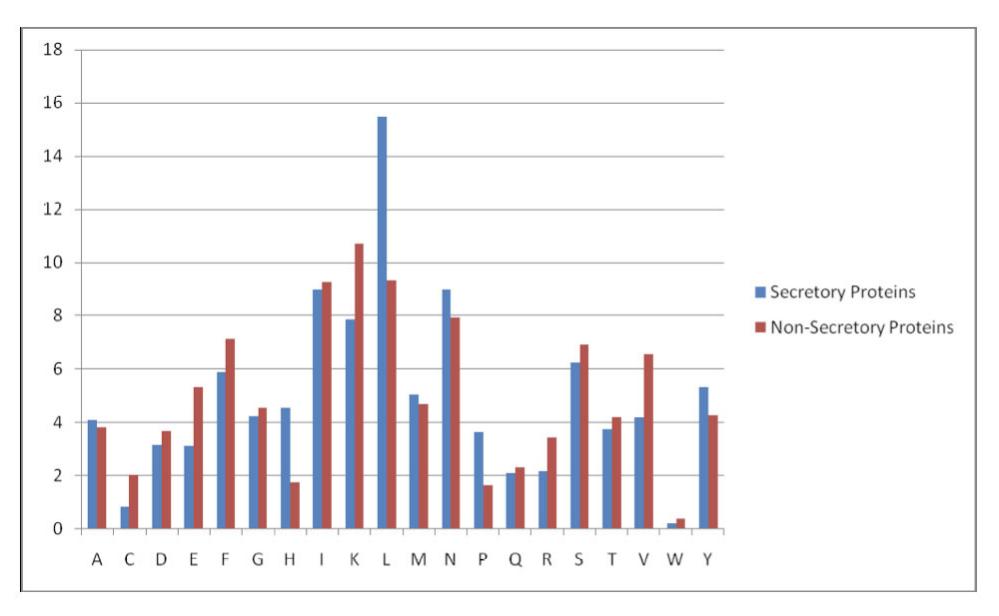
A Graph depicting the 25 amino acid of N-termini Amino acid composition of secretory and non-secretory proteins.

**Figure 3:**
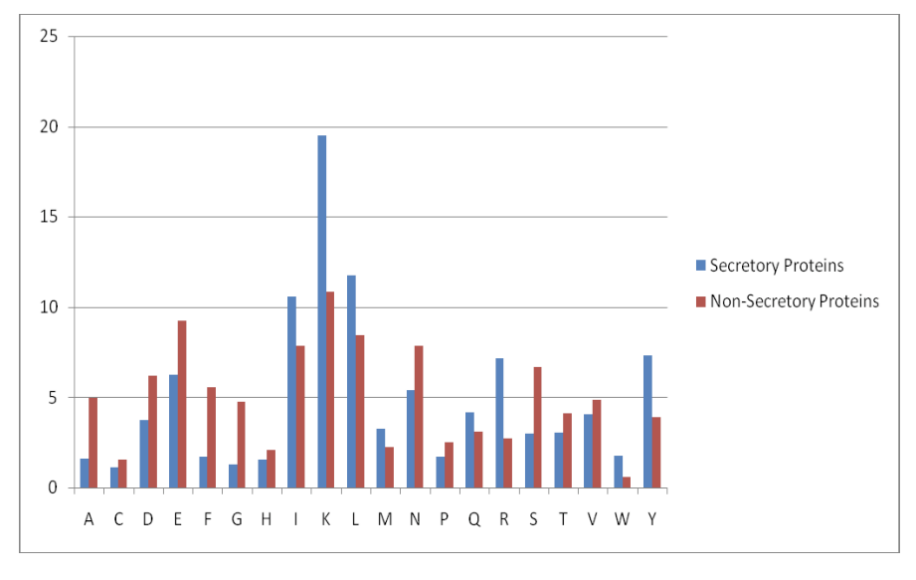
A Graph depicting the 25 amino acid of C-termini amino acid composition of secretory and non-secretory proteins.

**Figure 4:**
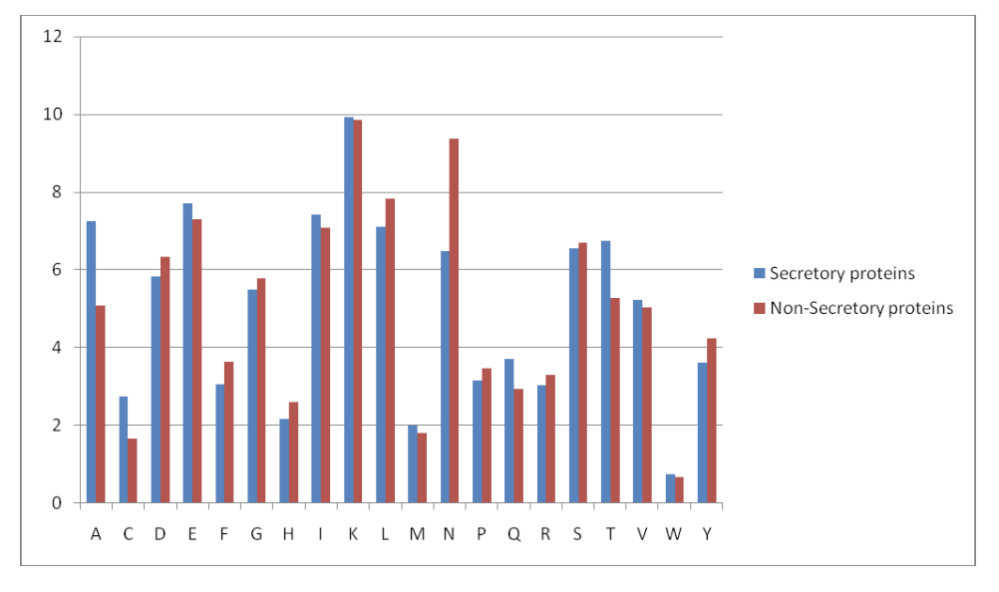
A Graph depicting the amino acid composition of middle part of secretory and non-secretory proteins.

We analyzed the amino acid composition of both secretory and non-secretory proteins. As shown in figure 1, the frequency of occurrence of amino acid alanine, cysteine, isoleucine, lysine, glutamine and threonine are higher in secretory proteins than non-secretory proteins, while composition of aspartic acid, phenylalanine, glycine are higher in non-secretory proteins than secretory proteins. There is a major difference of composition of asparagines in non-secretory protein (very high) than secretory protein. This means secretory proteins can be discriminated from non-secretory proteins based on their amino acid composition. It has been shown in previous studies that secretory proteins have signal sequence at N-termini). Thus it is important to compare composition of various parts of secretory and non-secretory proteins separately. As shown in Figure 2, N-terminal composition of two type of protein is quite different; magnitude of biasness is much higher than compositional biasness of whole protein. Similarly, composition of C-termini of secretory and non-secretory proteins is quite different (Figure 3). In comparison to it, difference in composition of central region of secretory and non secretory proteins was low (Figure 4).

### Composition based SVM models

It was observed that amino acid composition of secretory proteins was somewhat different from that of non-secretory proteins. Thus a SVM based classifier was developed using amino acid composition where amino acid composition was used as input vector of dimension 20. Different kernels and parameters of SVM were tried. The performance of our method on different thresholds is shown in Table 1. We got accuracy of around 84% with MCC 0.67 with nearly equal sensitivity and specificity. This model correctly predicts 76% secretory proteins at 96% specificity using RBF kernel. It has been observed that localization methods based on dipeptide composition perform better than amino acid composition based methods [4]. This is because dipeptides also provides information about local order of residues in addition to amino acid composition. For present study we developed SVM based method using dipeptide composition where dipeptide composition was used as input vector or dimension 400. As shown in Table 1, we obtained maximum accuracy of 86.45% with MCC 0.74 using dipeptides based SVM model. The SVM model based on dipeptides composition performed better than SVM model based on amino acid composition.

**Table 1:** The performance of SVM models using amino acids and dipeptides composition. The values in bold shows

**Thr**	**Amino acids composition**				**Dipeptides composition**			
	
	**Sn**	**Sp**	**Acc**	**MCC**	**Sn**	**Sp**	**Acc**	**MCC**
-1.0	94.84	27.60	61.35	0.30	98.81	0.80	50.00	0.00
-0.9	93.25	33.20	63.35	0.33	98.02	3.20	50.80	0.04
-0.8	92.86	37.60	65.34	0.37	96.83	4.00	50.60	0.02
-0.7	90.48	43.20	66.93	0.38	95.63	7.20	51.59	0.06
-0.6	89.29	48.00	68.73	0.41	94.44	12.40	53.59	0.12
-0.5	88.89	58.40	73.71	0.50	92.86	27.20	60.16	0.27
-0.4	87.70	65.60	76.69	0.55	88.49	49.60	69.12	0.41
-0.3	86.90	72.00	79.48	0.60	83.33	73.20	78.29	0.57
-0.2	85.71	78.00	81.87	0.64	**82.14**	**84.00**	**83.07**	**0.66**
-0.1	85.32	80.80	83.07	0.66	80.56	88.80	84.66	0.70
**0.0**	**83.33**	**84.00**	**83.67**	**0.67**	80.16	92.00	86.06	0.73
0.1	81.75	85.20	83.47	0.67	***78.57 ***	***94.40***	***86.45***	***0.74***
0.2	80.56	86.00	83.27	0.67	76.98	96.00	86.45	0.74
0.3	79.76	89.60	84.66	0.70	74.21	97.20	85.66	0.73
***0.4***	***78.97***	***92.40***	***85.66***	***0.72***	69.84	97.20	83.47	0.70
0.5	77.78	93.20	85.46	0.72	63.10	97.20	80.08	0.64
0.6	76.98	94.40	85.66	0.72	57.94	98.00	77.89	0.61
0.7	76.19	95.60	85.86	0.72	52.72	98.40	75.50	0.57
0.8	73.02	96.00	84.46	0.71	44.05	98.40	71.12	0.51
0.9	70.24	96.40	83.27	0.69	33.73	98.80	66.14	0.43
1.0	65.48	98.80	82.07	0.68	24.21	99.60	61.75	0.36

*Sn: Sensitivity; Sp: Specificity; Acc: Accuracy; MCC: Mathews Correlation Coefficient

### Split Amino Acid Composition

It has been observed that secretory proteins have signals either at N or C terminus. In order to utilize the compositional biasness in terminus of secretory and non-secretory proteins, we developed SVM models using split amino acid and dipeptides composition. As shown in Table 2, we got maximum accuracy 86.20% and 88.22% using split amino acid and dipeptides composition respectively. This is slightly better than accuracy achieved using whole composition. We found best performance with 25 N-terminal, 25 C-terminal and remaining protein.

**Table 2:** The performance of SVM models developed using amino acid and PSSM matrix composition.

**Thr**	**Split Amino acids composition**				**PSSM Matrix composition**			
	
	Sn*	**Sp**	**Acc**	**MCC**	**Sn**	**Sp**	**Acc**	**MCC**
-1.0	93.25	43.55	68.60	0.42	97.22	44.84	71.03	0.49
-0.9	93.25	50.00	71.80	0.48	95.63	58.73	77.18	0.58
-0.8	92.46	56.05	74.40	0.52	95.24	65.08	80.16	0.63
-0.7	91.27	59.68	75.60	0.54	94.05	70.24	82.14	0.66
-0.6	90.48	62.10	76.40	0.55	94.05	75.00	84.52	0.70
-0.5	87.70	64.11	76.00	0.53	92.86	84.13	88.49	0.77
-0.4	85.71	70.16	78.00	0.57	91.27	90.08	90.67	0.81
-0.3	84.92	73.39	79.20	0.59	**90.08**	**91.27**	**90.67**	**0.81**
-0.2	83.73	78.63	81.20	0.62	90.08	92.86	91.47	0.83
-0.1	**83.33**	**82.26**	**82.80**	0.66	89.29	94.05	91.67	0.83
0.0	81.75	87.50	84.60	0.69	89.29	94.84	92.06	0.84
***0.1***	***79.37***	***93.55***	***86.40***	***0.74***	88.89	95.24	92.06	0.84
0.2	77.38	94.76	86.00	0.73	***88.49***	***96.83***	***92.66***	***0.86***
0.3	76.19	96.37	86.20	0.74	87.30	97.22	92.26	0.85
0.4	73.81	97.98	85.80	0.74	85.71	97.22	91.47	0.83
0.5	71.43	97.98	84.60	0.72	85.32	98.41	91.87	0.84
0.6	69.84	97.98	83.80	0.71	84.92	98.81	91.87	0.85
0.7	68.25	97.98	83.00	0.69	83.73	98.81	91.27	0.83
0.8	65.87	97.98	81.80	0.67	82.94	99.21	91.07	0.83
0.9	63.10	98.39	80.60	0.66	80.16	99.21	89.68	0.81
1.0	58.73	99.60	79.00	0.64	73.41	100.00	86.71	0.76

*Sn: Sensitivity; Sp: Specificity; Acc: Accuracy; MCC: Mathews Correlation Coefficient

### PSSM based SVM models

In past, multiple sequence alignment information in form of position specific scoring matrix (PSSM) has been used for developing methods [15-17]. In this study, PSSM has been used for predicting secretory proteins. First we created PSSM profile for each protein using PSI-BLAST search against nr database with three iterations, at cut-off 0.01. Secondly, we computed a vector of dimension of 400 from PSSM matrix. Finally a SVM model was developed using PSSM and we achieved maximum accuracy of 92.66% with MCC 0.86. In addition this model was able to correctly predict 73% secretory proteins at specificity 100%. This clearly demonstrates that PSSM provide more information than single sequence and is useful for predicting secretory proteins.

### Pseudo amino acid composition (PseAAC)

In past PseAAC has been widely used for classifying the proteins and subcellular localization methods. Thus we also tried to develop SVM models using simple PseAAC. In this study we have computed pseudo amino acid composition using PseAAC [18,19]. We found that the performance of PseAAC based model is better than model based on amino acids or dipeptides composition. However, performance is poor than our PSSM based model (Table 3). We tried two characters Hydrophobicity and PI and performance of which was nearly same.

**Table 3:** The performance of SVM models developed using pseudo amino acid composition (PseAAC).

**Thr**	**Pseudo Amino acid composition (hydrophobicity)**				**Pseudo Amino acid composition (pI at 25^0 ^C.)**			
	
	Sn*	**Sp**	**Acc**	**MCC**	**Sn**	**Sp**	**Acc**	**MCC**
-1.0	92.86	58.17	75.55	0.54	96.03	44.44	70.24	0.47
-0.9	92.06	66.14	79.13	0.60	94.44	50.79	72.62	0.50
-0.8	91.67	72.91	82.31	0.66	93.25	59.13	76.19	0.56
-0.7	90.08	74.50	82.31	0.65	92.46	61.51	76.98	0.57
-0.6	89.29	77.29	83.30	0.67	91.67	65.08	78.37	0.59
-0.5	88.89	82.47	85.69	0.72	91.67	69.44	80.56	0.63
-0.4	88.10	85.26	86.68	0.73	90.48	75.40	82.94	0.67
-0.3	86.11	88.45	87.28	0.75	89.29	78.57	83.93	0.68
-0.2	85.32	90.04	87.67	0.75	89.29	80.16	84.72	0.70
-0.1	85.32	92.83	89.07	0.78	88.89	84.13	86.51	0.73
0.0	83.73	94.02	88.87	0.78	87.70	86.90	87.30	0.75
0.1	82.94	95.22	89.07	0.79	86.90	88.49	87.70	0.75
0.2	81.75	95.62	88.67	0.78	86.11	90.48	88.29	0.77
0.3	81.75	96.41	89.07	0.79	84.13	91.67	87.90	0.76
0.4	81.35	96.81	89.07	0.79	83.33	92.46	87.90	0.76
0.5	80.56	96.81	88.67	0.78	82.54	94.44	88.49	0.78
0.6	78.57	98.41	88.47	0.79	81.35	95.24	88.29	0.77
0.7	76.98	98.41	87.67	0.77	79.76	95.63	87.70	0.76
0.8	73.02	98.41	85.69	0.74	78.17	97.22	87.70	0.77
0.9	68.25	98.80	83.50	0.70	73.02	97.22	85.12	0.72
1.0	62.30	98.80	80.52	0.66	67.06	98.02	82.54	0.68

#### Benchmarking

In order to compare performance of our method with existing methods, we predicted proteins used in this study using existing methods. Firstly, we applied PlasmoHT that is based on motif and specially developed for predicting secretory proteins in Plasmodium [8]. In order to use PlasmoHT one need to provide PlasmoDB ID, as all proteins in our dataset are not from PlasmoDB database so it could not be applied on all the proteins [20]. This method correctly predicted 146 out of 246 secretory proteins (six proteins do not have Plasmodb ID). PlasmoHT fails to predict 100 secretory proteins since all secretory proteins do not have conserved signal motif. It also correctly predicted 54 out of 55 non-secretory proteins obtained from PlasmoDB. It was not possible to apply PlasmoHT directly on 197 non-secretory proteins obtained from Swiss-Prot as PlasmoHT need PlasmoDB ID. Thus, we manually examined the Swiss-Prot entries and found 53 entries have ORFname (matches with PlasmoDB ID) in field Gene name. Six Swiss-Prot entries out of 53 have two or more than two ORF names (it not necessary that every protein will have one ORF). We examined 47 proteins for which single PlasmoDB ID was available and found that two proteins had PlasmoHT motif. It means PlasmoHT correctly predicted 45 out of 47 non-secretory proteins. In total PlasmoHT correctly predicted 99 out 102 non-secretory proteins. We were not able to locate PlasmoDB ID for all proteins extracted from Swiss-Prot, which may be due to number of reasons i.e. modified form of protein; mutated proteins or protein fragments. Secondly we applied commonly used method for predicting secretory proteins TargetP on our dataset, it correctly predicted 163 out of 251 secretory proteins (unable to predict one protein due to its large size) [3]. It also correctly predicted 160 out of 252 non-secretory proteins. In summary, we achieved sensitivity 64.94%, specificity 63.49% and accuracy 64.21% using TargetP on our dataset. Thirdly, we evaluated performance of two commonly used subcellular localization methods PA-SUB and WoLF PSORT on our dataset [21,22]. PA-SUB first extract the features of similar sequences to query sequence from Swiss-Prot then it uses machine learning model for predicting subcellular location of query protein [21]. WoLF PSORT converts protein amino acid sequences into numerical localization features; based on sorting signals, amino acid composition and functional motifs such as DNA-binding motifs. After conversion, a simple *k*-nearest neighbor classifier is used for prediction [22]. Performance of both methods is shown in Table 4, both methods fail to predict secretory proteins of P. *Falciparum*.

**Table 4:** The prediction of location of proteins in our datasets using various methods. Our dataset have 252 secretory and 252 non-secretory. The values in bracket shows total number of proteins on which a method was applied.

**Subcellular Location**	**PA-SUB**		**WoLF PSORT**		**TargetP**		**PlasmoHT**	
	
	**NSec^a ^(250)**	**Sec^b ^(252)**	**NSec (250)**	**Sec (250)**	**NSec (252)**	**Sec (251)**	**Nsec (102)**	**Sec (246)**
**Nuclear**	36	11	26	23	--	--	--	--
**Plasma Membrane**	0	1	20	67	--	--	--	--
**Extracellular**	48	12	36	22	92	163	3	146
**Cytosol**	89	15	98	48	--	--	--	--
**Others**	42	12	70	82	--	--	--	--
**NSec**	--	--	--	--	160	89	98	100
**No Prediction**	45	206	--	--	--	--	--	--

^a^Nsec: Non-secretory proteins; ^b^Sec: Secretory proteins

## Discussion

*Plasmodium falciparum *during its asexual stage within the host erythrocyte remodels the host cell displaying several dramatic changes, which affects membrane rigidity surface antigenicity and permeability. These changes aid in the pathogenesis and also help the parasite survival within null host cell by nutrient acquisition [23]. It has been estimated that an array of parasite derived antigens are expressed on infected cell membrane [24,25]. However, only a few protein such as PfEMP-1, rifin and stevor family proteins have been conclusively proven to be on the surface of infected erythrocyte membrane. The search of parasite derived proteins within the host cell and infected membrane surface remains one of the most warranted areas in malaria research for understanding the pathogenesis of disease, and to find out potent vaccine candidate molecule. Recently, two independent groups [7,8] have done *in silico *prediction of proteins exported into the host erythrocyte (a 'secretome') based on the *Plasmodium *export element (PEXEL) [7] and the vacuolar transport signal (VTS) [8] motifs. These motifs were identified by bioinformatic analysis of aligned N-terminal sequences from proteins known to be exported from the parasitophorous vacuole (PV) into the erythrocyte. Whereas Hiller *et al*. [8] used reiterative alignments to search for motif while Marti *et al.*[7] used a search protocol based on the presence of signal sequence (SS) on exon I. Both reported motifs contains a short stretch of alternating charged and hydrophobic amino acids separated by uncharged amino acids located a short distance downstream of the SS. Functional role of PEXEL/VTS motif has been demonstrated by GFP fusion with SS followed by live fluorescence imaging and mutational analysis of PEXELl/VTS motif. However, PEXEL/VTS dependent protein trafficking cannot be typified due to over and possible incorrect timed expression of chimeric GFP fusion protein [9]. Moreover RESA-GFP chimera containing PEXEL/VTS was reported to be mistargeted to lumen of parasitovorous vacuole [26]. Besides well known exported proteins the predicted protein also includes several proteins for which export into the erythrocyte had not previously been shown, including several heat-shock proteins, kinases, phosphatases and putative transporters [8]. But one of the major limitation of the prediction based on PEXEL/VTS motif is that it could not predict proteins lacking PEXEL/VTS motif but experimentally demonstrated to be exported into the erythrocyte, such as *P. falciparum *skeleton-binding protein (PfSBP), membrane-associated histidine-rich protein (MAHRP) and coat protein (COP)II, all of which seem to be associated with vesicles and/or Maurer's clefts [27]. Moreover, the above-mentioned motif based methods gets setback in case of members of the *vir *supergene family (homologues *vir*/*bir*/*gir*), proteins predicted to be expressed on the erythrocyte surface [28] since none of these have SS or PEXEL/VTS motifs. Unlike many parasite-encoded proteins exported into the erythrocyte, PfEMP1 lacks an SS. Although both groups were able to identify a conserved sequence with biophysical characteristics similar to those of the more classical PEXEL/VTS, by creating mini-PfEMP1 reporter constructs consisting of their respective PfEMP1 PEXEL/VTS motif, GFP or YFP and the conserved C terminus of PfEMP1 (including the TM). But the location of PEXEL/VTS in PfEMP1 is contradicting. Marti *et al. *[7] described the motif to be located ~16–32 amino acids in from the N terminus, whereas Hiller *et al. *[8] reported the motif to be ~300 amino acids further downstream, within a semi-conserved Duffy-binding-like (DBL) domain. This discrepancy in the location of PEXEL/VTS motifs points to an ambiguity for the existence of identical and universal motif for exported proteins predicted to be exported into erythrocyte. Although Florens et al. [29] have predicted about 36 hypothetical proteins of the parasite to be located on infected erythrocyte surface using multidimensional protein technology (MudPIT), but their groups have not proposed any universal method for prediction. Nevertheless, the literature strongly advocates the existence of multiple pathways that are cumulatively responsible for the export of parasite proteins in erythrocytes [9].

## Conclusion

The bioinformatics approach used in this study is standard approach, which is commonly used for predicting subcellular localization of proteins. In addition evolutionary information in form of PSSM has been used first time for predicting secretory proteins in malaria parasite. Our model is equally applicable to wide range of secretory proteins where most of method fails. One of the major advantage of method describes in this study is that it is based on complete sequence rather than on small region/motif. The server developed for predicting secretory proteins will be very useful for researchers working in the field of malaria.

## Methods

### Datasets

#### Secretory or positive dataset

From the literature we collected total 267 secretory proteins consisting of 208 secretory proteins (119 Rifins, 22 Stevors, 67 PfEMP1); 6 experimentally proven proteins (PF10_0159, PFE0040c, PFB0100c, PFB0095c, AAD31511, AAC47454) [7]. Another set of 3 experimentally proved secretory proteins (PFD1175w, PFD1170c, PFB0100c) [8]; more 7 proteins (PFI1755c, PFE0055c, PFI1780w, PFE0360c, PF10_0321, PF14_0607, PFE0355c); 4 REX proteins (PFI1740c, PFI1755c, PFI1760w, PFI1735c); 2 PIESPs (PFC0435c, PFE0060w); clag9 (PFI1730w); Sbp1 (PFE0065w) and 35 maurer's cleft associated proteins [30-32]. These all sum up to 267 secretory protein. We got 252 non-redundant secretory proteins after removing redundant proteins using program PROSET [33].

#### Non-secretory or Negative dataset

Selection of negative dataset is always a challenge. We normally prefer to get negative dataset from manually annotated database Swiss-Prot. In this study, we extracted non-secretary protein from Swiss-Prot using SRS with query "*Plasmodium Falciparum *(Organism) but not Secreted (comment)". This way we got 197 non-secretory proteins and we required 252 non-secretory proteins in order to make both secretory and non-secretory proteins equal. Thus we used another database PlasmoDB to extract remaining 55 non-secretory proteins. We extracted nuclear proteins from PlasmoDB and randomly picked up 55 proteins from ~300 nuclear proteins. This way we got 252 non-secretory proteins from two sources (197 Swiss-Prot and 55 PlasmoDB). We extracted equal number of negative examples in order to evaluate performance from single parameter like accuracy and MCC.

#### Composition

The aim of calculating composition of proteins is to transform the variable length of protein sequence to fixed length feature vectors. This is important to classify proteins using machine-learning techniques because they required fixed length pattern. The information of proteins can be encapsulated to a vector of 20 dimensions using amino acid composition of the protein [4,5]. In addition to amino acid composition, dipeptide composition was also calculated which present protein by a vector of 400 dimensions. The advantage of dipeptide composition over amino acid composition is that it encapsulates information about the fraction of amino acids as well as their local order.

#### Split Amino Acid Composition (SAAC)

We split protein in three parts and compute composition of each part of protein separately. This way we created a vector of a dimension 60 (3 × 20) instead of 20 in case of amino acid composition [34]. In SAAC each protein was divided into three parts: (i) 25 amino acids of the N terminus, (ii) 25 amino acids of the C terminus, and (iii) remaining protein length after removing 25 amino acids from N- and C-terminus. The rationale behind using SAAC is that difference in composition of secretory and non-secretory proteins is more prominent if terminal residues are compared separately instead with whole protein. It is known that most of secretory proteins have signals at N-terminal. The advantage of SAAC over standard amino acid composition is that it provides greater weight to proteins that have a signal at either the N or C.

#### Multiple sequence alignment in form of PSSM profiles

In the present study multiple sequence alignment in the form of PSSM has been used for predicting secretory proteins. Recently number of study used PSSM profile composition for developing prediction methods; for example MemType-2L, EzyPred, Tbpred, DNAbinder, SRTpred used for predicting membrane, enzyme class, subcellular localization of M. Tuberculosis, DNA binding and secretory proteins respectively [34-38]. The PSSM for each sequence was generated by performing PSI-BLAST search against 'nr' database using three iterations with cut off e-value 0.001. For a sequence of length *N *residues, PSSM is represented by an *N *× 20 matrix (dummy residue 'X' is ignored) [29,30]. Each element of this matrix, *m *[*i, j*], provides information on evolutionary conservation of residue type *j *at sequence position *i*. We coverted this matrix in a vector of dimension 400, by computing composition of occurrences of each type of amino acid corresponding to each type of amino acids in protein sequence. It means for each column we will have 20 values instead of one [34,37]. Every element in this input vector was subsequently divided by the length of the sequence and then scaled to the range of 0–1 by using the standard sigmoid function as described by Rashid et al. [34]. The resultant matrix with 400 elements was used as input feature for SVM.

#### Support Vector Machine

In this study we implemented SVM using SVM_light package which allows choosing number of parameters and kernels (e.g. linear, polynomial, radial basis function, sigmoid) or any user-defined kernel. The selection of kernel is very important in SVM, which is analogous to choosing architecture in ANN. In this study, learning was carried out using three kernels linear, polynomial and RBF.

#### Evaluation

The performance of any prediction algorithm is often checked by jack-knife tests or cross-validations. In current study the performance of all the methods and models was evaluated using 5-fold cross-validation in which the dataset was randomly divided into five equal sets, out of which four sets were used for training and the remaining one for testing. This procedure was repeated five times in such a way that each set is tested once. The final performance was calculated by averaging over all five sets. The performance of our method was computed by using following standard parameters.

**(a) Sensitivity or coverage of positive examples: **It is percent of secretory proteins correctly predicted secretory.

$$\text{Sensitivity (Sn)}=\frac{\text{TP}}{\text{TP }+\text{ FN}}\times 100$$

**(b) Specificity or coverage of negative examples: **It is percent of non-secretory proteins correctly predicted non-secretory.

$$\text{Specificity (Sp)}=\frac{\text{TN}}{\text{TN }+\text{ FP}}\times 100$$

**(c) Accuracy: **It is percentage of correctly predicted proteins (secretory and non-secretory proteins).

$$\text{Accuracy (Acc)}=\frac{\text{TP}+\text{TN}}{\text{TP}+\text{TN}+\text{FP}+\text{FN}}\times 100$$

**(d) Mathew's correlation coefficient (MCC): **It is considered to be the most robust parameter of any class prediction method. MCC equal to 1 is regarded as perfect prediction while 0 for completely random prediction.

$$\text{MCC}=\frac{\text{(TP}\times \text{TN) -(FP}\times \text{FN)}}{\sqrt{\text{(TP}+\text{FP)(TP}+\text{FN)(TN}+\text{FP)(TN}+\text{FN)}}}$$

where TP and TN are truly or correctly predicted secretory and non-secretory proteins. FP and FN are wrongly predicted secretory and non-secretory proteins.

## Availability and requirements

PSEApred: 

## Authors' contributions

RV developed computer programs, implement SVM and developed the web server. AT and SK collected data, annotated proteins and validated results. GPSR and GV conceived the idea, coordinated the project and refined the drafted manuscript. GPSR guided its conception and helped in interpretation of data and gave overall supervision to the project. All authors read and approved the final manuscript.
